# Supplementary material for: Optical Genome Mapping for Comprehensive Assessment of Chromosomal Aberrations and Discovery of New Fusion Genes in Pediatric B-Acute Lymphoblastic Leukemia
Source: Cancers (Basel). 2022 Dec 21;15(1):35. doi: 10.3390/cancers15010035 (PMC9817688; doi:10.3390/cancers15010035)

A

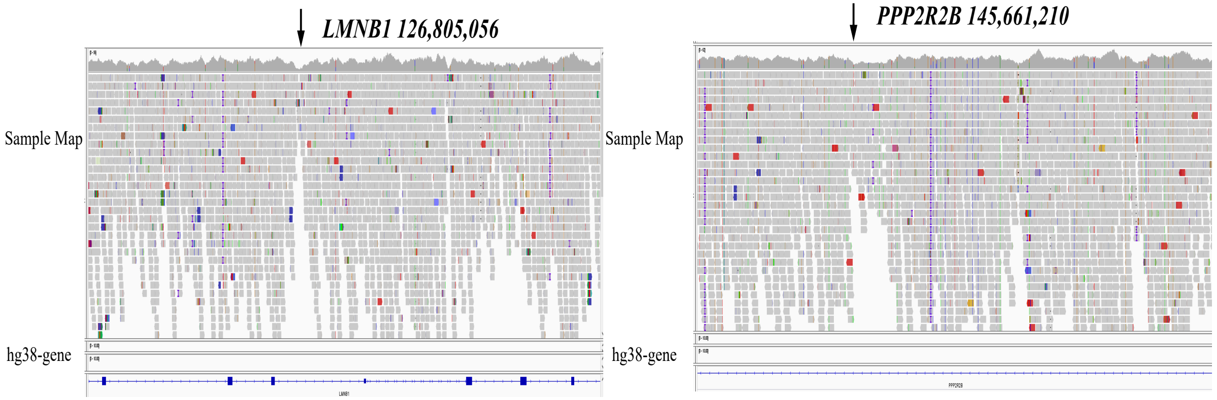

Source	Chr	Start	End	SVType	SVLen	Check	Gene_Fusion
OGM	chr5	126,803,710	146,674,262	DEL	19,870,552		LMNB1-PPP2R2B
WGS	Chr5	126,805,056	146,661,210	DEL	19,856,154	Ture	LMNB1-PPP2R2B

B

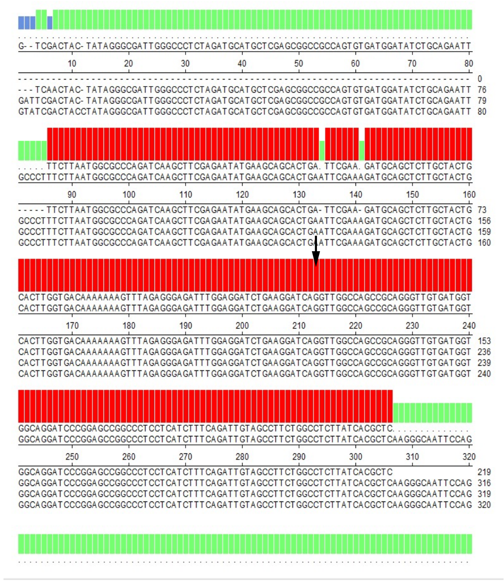

AGCTGCTCCTCAAGCTATGCTAAGAAGGAATCTGA***TCTTAA***
TGGCGCCCAGATCAAGCTTCGAGAATATGAAGCAGCACTG
AATTCGAAAGATGCAGCTCTTGCTACTGCACTTGGTGACA
AAAAAAGTTTAGAGGGAGATTTGGAGGATCTGAAGGATCA
GGTTGGCCAGCCGCAGGGTTGTGATGGTGGCAGGATCCCG
GAGCCGGCCCTCCTCATCTTTCAGATTGTAGCCT***TCTGGCC***
TCTTATCACGCTCGCTGACTTTCCACAGCTTCACAGTTTTAT
CATTAGTAGACAGAAGAAAGTAAGCTGCATTCTGCTGGG

(Product size 220bp)

C

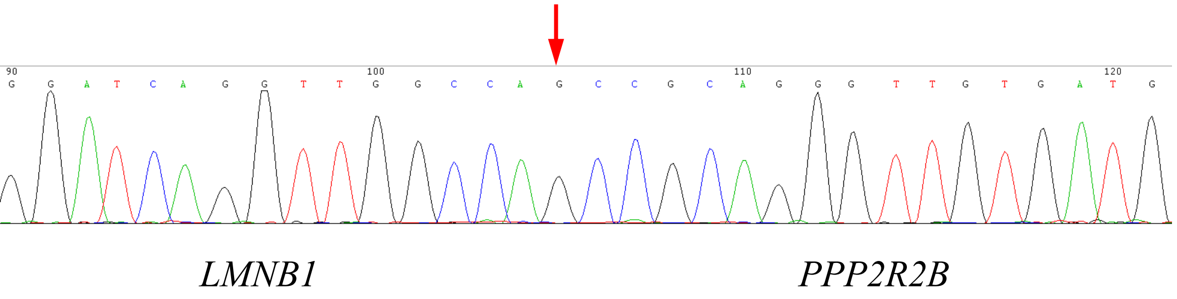

D

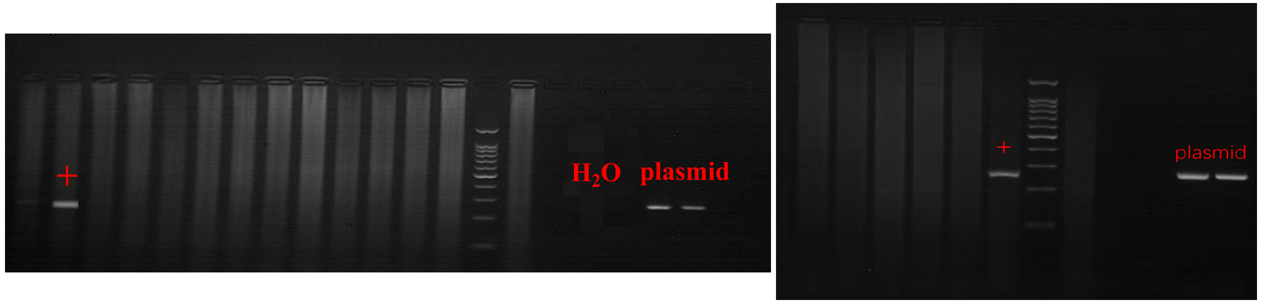

Supplement: Supplementary file 1 [file cancers-15-00035-s001.zip › Supplementary files/Supplementary Figure 3.pdf]
