# Supplementary figures and images for: Optical Genome Mapping for Comprehensive Assessment of Chromosomal Aberrations and Discovery of New Fusion Genes in Pediatric B-Acute Lymphoblastic Leukemia
Source: Cancers (Basel). 2022 Dec 21;15(1):35. doi: 10.3390/cancers15010035 (PMC9817688; doi:10.3390/cancers15010035)

A

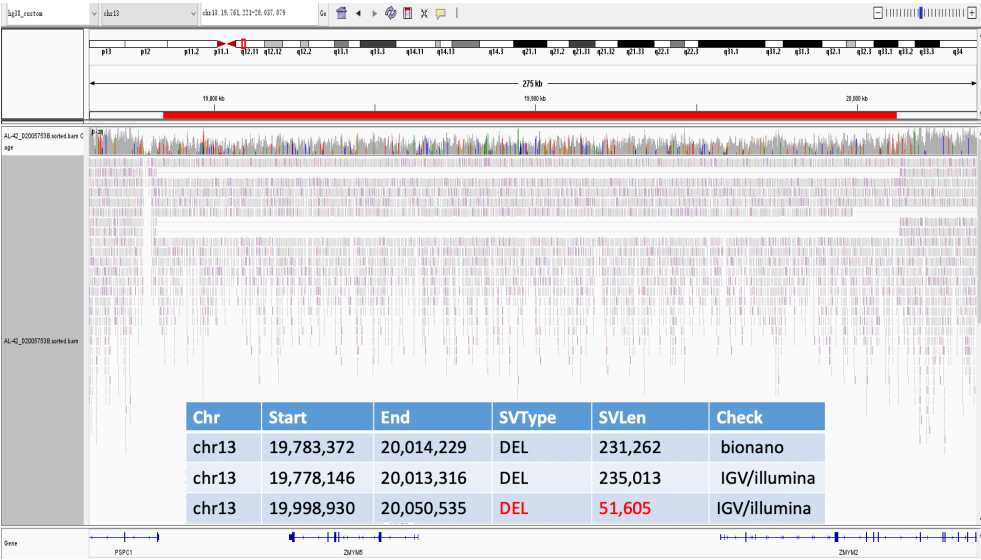

B

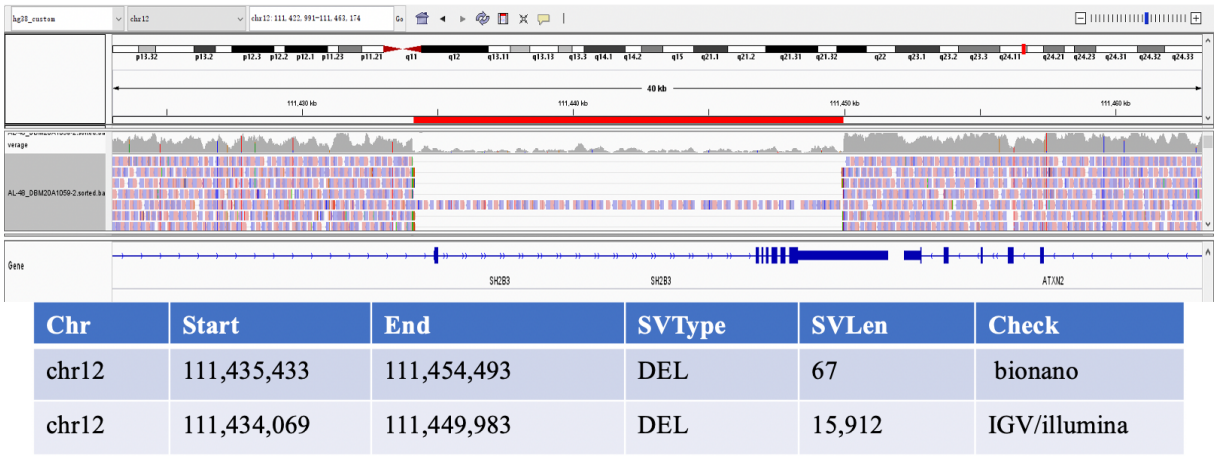

C

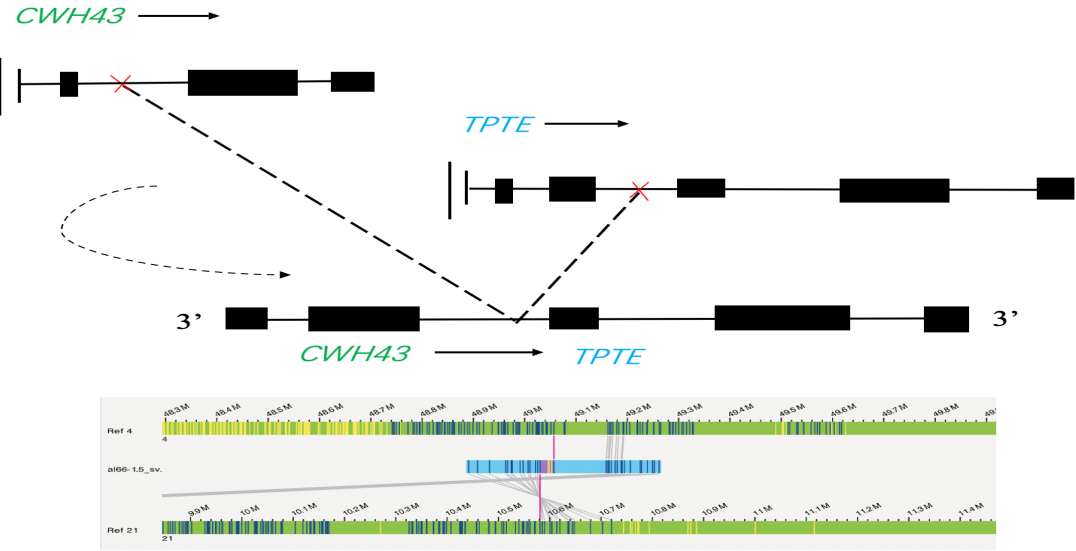

Supplement: Supplementary file 1 [file cancers-15-00035-s001.zip › Supplementary files/Supplementary Figure. 1.pdf]
