# Supplementary material for: Optical Genome Mapping for Comprehensive Assessment of Chromosomal Aberrations and Discovery of New Fusion Genes in Pediatric B-Acute Lymphoblastic Leukemia
Source: Cancers (Basel). 2022 Dec 21;15(1):35. doi: 10.3390/cancers15010035 (PMC9817688; doi:10.3390/cancers15010035)

A

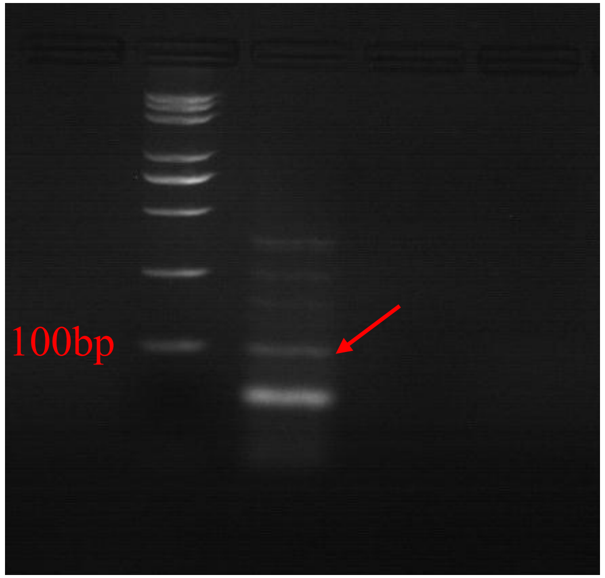

aactttgtcagcgagcaagaacaatgcc
aggaggtctggagaaaacgtgtcatca
gtgcatttctaaaatcgccagcaatggaa
ccatcactgttgctggaggcacctgacaa
atcctagcgaatttttggagcatctccacc
cagg

F:ACAATGCCAGGAGGTCTGGA
R:AGGATTTGTCAGGTGCCTCC
Product size 98bp

B

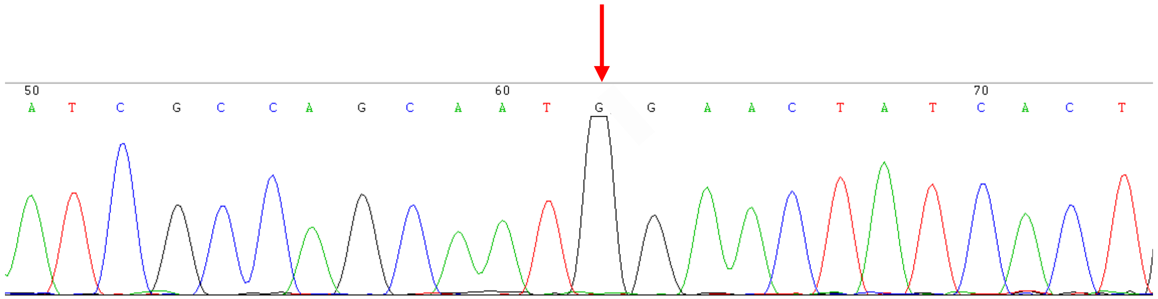

C

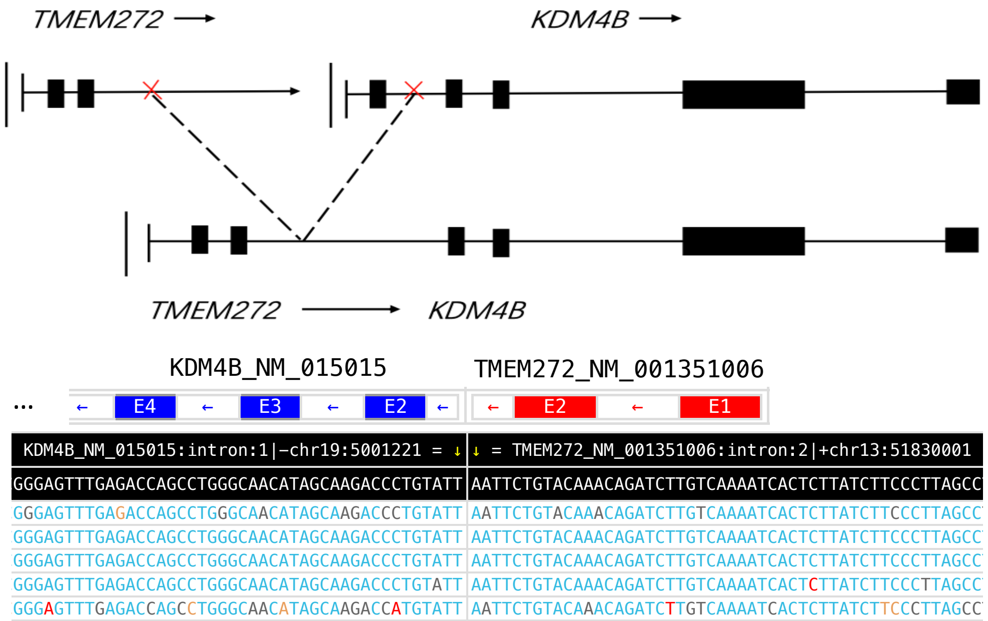

Supplement: Supplementary file 1 [file Supplmentary Figure 2.pdf › Supplementary files/Supplmentary Figure 2.pdf]
